# Forecasting the progression of human civilization on the Kardashev Scale through 2060 with a machine learning approach

**DOI:** 10.1038/s41598-023-38351-y

**Published:** 2023-07-12

**Authors:** Antong Zhang, Jiani Yang, Yangcheng Luo, Siteng Fan

**Affiliations:** 1 Division of Geological and Planetary Sciences, California Institute of Technology, Pasadena, CA 91125 USA; 2 LMD/IPSL, Sorbonne Université, PSL Research University, École Normale Supérieure, École Polytechnique, CNRS, 75005 Paris, France

**Keywords:** Environmental social sciences, Mathematics and computing

## Abstract

Energy has been propelling the development of human civilization for millennia. Humanity presently stands at Type 0.7276 on the Kardashev Scale, which was proposed to quantify the relationship between energy consumption and the development of civilizations. However, current predictions of human civilization remain underdeveloped and energy consumption models are oversimplified. In order to improve the precision of the prediction, we use machine learning models random forest and autoregressive integrated moving average to simulate and predict energy consumption on a global scale and the position of humanity on the Kardashev Scale through 2060. The result suggests that global energy consumption is expected to reach ~ 887 EJ in 2060, and humanity will become a Type 0.7449 civilization. Additionally, the potential energy segmentation changes before 2060 and the influence of the advent of nuclear fusion are discussed. We conclude that if energy strategies and technologies remain in the present course, it may take human civilization millennia to become a Type 1 civilization. The machine learning tool we develop significantly improves the previous projection of the Kardashev Scale, which is critical in the context of civilization development.

## Introduction

Throughout the history of human civilization, energy has been holding an imperative role in humanity’s progress^1^. Especially in the past few centuries, innovations in the harnessing of power have catalyzed humanity’s rapid growth^2^. Energy remains a key driver of human development^3^, with each revolution in industry and agriculture highlighting human’s reliance on it. Revolution in the eighteenth century was a turning point. The development of steam engines powered by fossil fuels led to significant technological progress^4^. Electricity has then opened new possibilities for the future^5^. Humanity has grown at a compound annual rate of 2.43% from 1965 to 2020, demonstrating our continued increasing demand for and consumption of energy^6,7^. However, the pace at which human being can progress as a civilization in the future remains uncertain.

While mankind was establishing its identity in the universe, insatiable human curiosity over the realm of civilization peaked in the 1960s^8^, which led to deeper cogitation of the concept of civilization. Providing that some of the extraterrestrial civilizations are highly likely million years more advanced than mankind, Soviet astrophysicist Nikolai Kardashev proposed a scale to classifies a civilization’s technological development based on its energy consumption^9^, which was later known as the Kardashev Scale. The scale initially categorized civilizations into three types. Type 1 is known as the planetary civilization, which features the capability of harnessing and utilizing all forms of energies that can be reached on the host planet, such as wind, solar, and geothermal power; Type 2 and 3, known as the stellar and galactic civilizations, respectively, are capable of extracting and utilizing all energy created by their respective systems^9^. Yet, such a scale proved lackluster in the quantitative presentation of the civilization types. Subsequently, Carl Sagan furthered the Kardashev Scale with data extrapolation, and proposed a continuous function quantifying the Kardashev Scale in index K^10^

$$K=\frac{\mathrm{log}P-6}{10}$$

where P represents the energy consumption rate in Watt. Sagan estimated that, by approximation, a Type 2 civilization should meet an energy consumption rate of 10^26^ W and a Type 3 civilization of 10^36^ W, both of which represent the cumulative energy output of their respective systems. Extrapolating these two values, he suggested a Type 1 civilization to have an energy consumption rate of 10^16^ W.

There is much progress to be desired before humanity is able to acquire the energy capacity to complete its first stride on the Kardashev Scale. Currently, mankind is measured on the scale at K =  ~ 0.7276^7^. On the bright side, the bloom of promising new technologies being developed and others that are yet to come betoken rapid progress. A potential energy source that may behoove mankind is nuclear fusion, which could generate nearly limitless, pollution-free energy^11,12^.

The development of the Kardashev Scale and the Drake equation^13^ received continuous interests in the search for technologically developed extraterrestrial intelligence with the exception of planetary boundaries^14^. Nevertheless, despite considerable work^15,16^ in energy forecasting and scenario planning over the past few decades, predicting the prospects of our civilization on the Kardashev Scale remains an underdeveloped area^17^. A recent study projected the human civilization to reach Type 1 in the mid-twenty-fourth century using linear regression^18^. However, it oversimplified the complex relationship between total energy consumption and various contributing factors, and its assumption of a linear increase of K index with year does not agree with historical data, which instead suggests a logarithmic trend. To arrive at a more precise and reliable prediction of humanity’s future energy consumption and progress on the Kardashev Scale, methods with more comprehensive consideration of influencing factors are needed.

Artificial intelligence and machine learning (ML) approaches have revolutionized energy forecasting, enabling the identification of patterns and hidden information in historical data for making predictions^19^. Among them, deep neural networks have been widely used to predict energy consumption^20^ due to their outstanding performance in interpreting hidden layers^21^, but difficulties remain in evaluating the influence of individual input variables^22^. On the contrary, being an ensemble learning algorithm for classification, the random forest (RF) model is able to derive reliable results with the capability of providing a ranking of input parameters based on their relative significance and can handle high nonlinearity^23^. This makes it particularly suited for training with multi-dimensional complex data in energy consumption predictions^24,25^. By actively incorporating the role of each parameter into the forecasting process, RF enables comprehension of the contributors of future energy changes. Given these advantages, RF is used in this work to find the non-linear relationship between energy consumption and influencing factors, capitalizing on its strengths. To aid gaps in the data considered in this work, another machine learning technique, the autoregressive integrated moving average^26^ (ARIMA), is utilized in the model to forecast total energy consumption and the Kardashev index, K, of human civilization from now until 2060. The projection is made with a new forecasting strategy supported by datasets of economic, climate, and planetary boundary parameters. A hypothesis about the potential advent of nuclear fusion is also evaluated. This study offers a broad view of future energy developments before humanity enters a series of energy transformations.

## Results

### Major contributors to energy consumption

**Figure 1:**
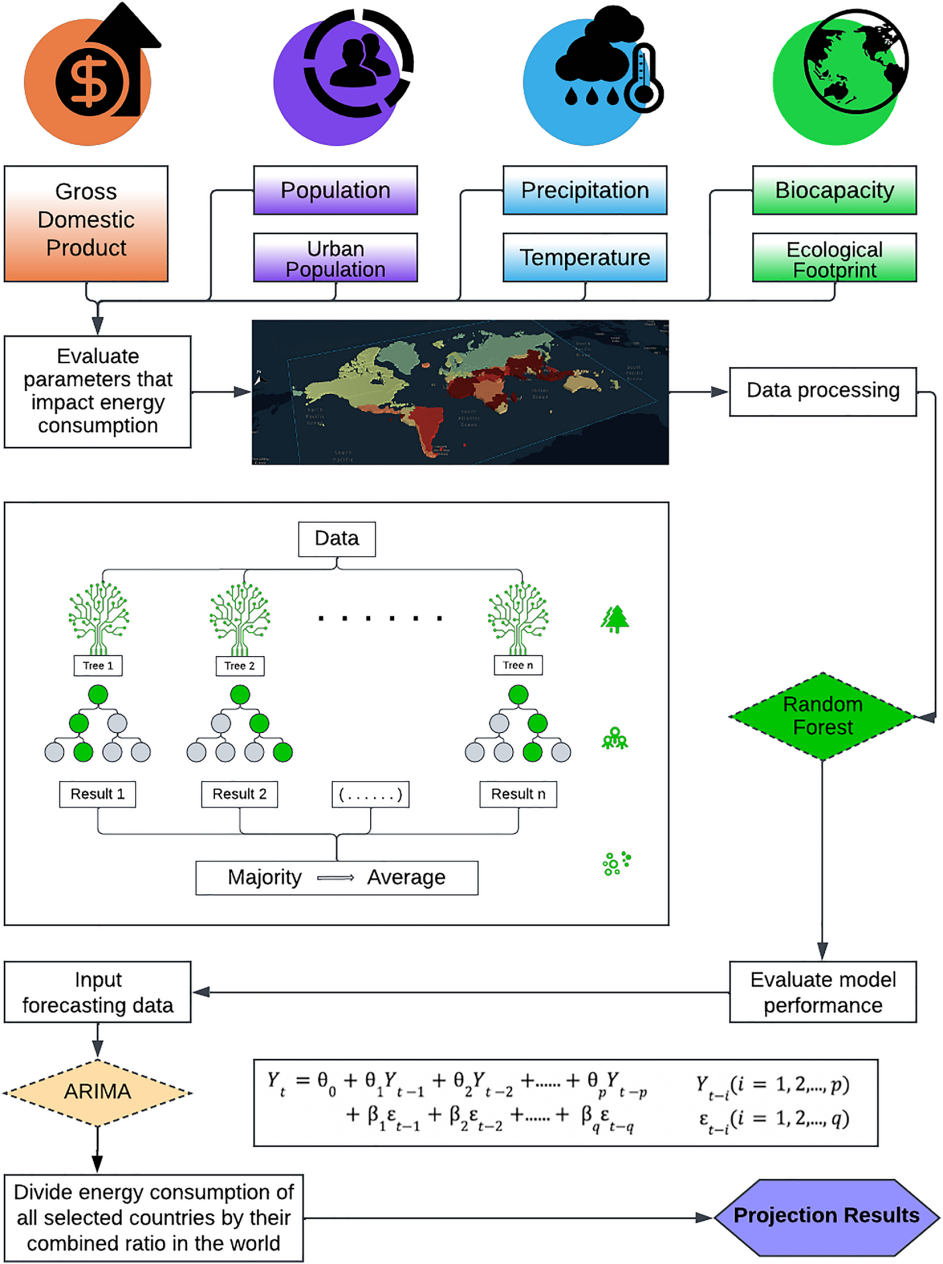
Experiment procedures with variable charts and demonstration of applied machine learning models. The input parameters used to forecast energy consumption are listed and categorized. Each node represents a main step in the experiment procedures, and the arrows connect the steps. The RF model’s general form is visualized and the equation for the ARIMA model is presented.

**Figure 2:**
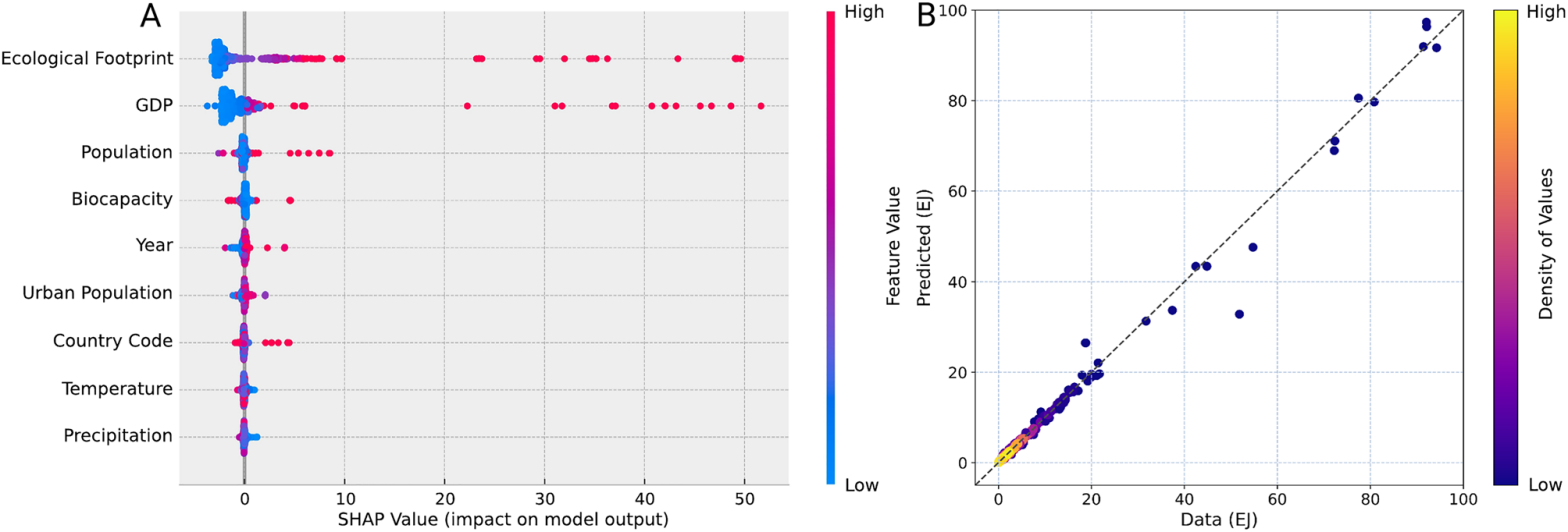
Impact of major input parameters on the model output and performance of the RF model. (**A**) The selected factors’ impacts on the total energy consumption are measured by their SHAP values. The color of each individual dot represents the value of that feature. (**B**) The model projection is compared with the validation dataset, which is extracted as 20% of the input data. A dashed line with a unit slope is shown for reference.

RF is utilized in this study to estimate the total energy consumption in the world. The model captures the nonlinearity among global economy, climate, and planetary boundary variables (Fig. 1). For economic indicators, gross domestic product (GDP), population, and urban population are selected as inputs; in terms of climate factors, temperature and precipitation are included as they directly impact human demand for energy; planetary boundary describes nine variables of high importance to habitability of Earth, and here we use ecological footprint and biocapacity to represent the human interaction with Earth’s biological resources. Shapley additive explanations (SHAP) is used to estimate and to illustrate the impact of each variable on the total energy consumption (Fig. 2A). The SHAP value breaks down a projection to demonstrate the impact of each parameter through utilizing the traditional Shapley values, which is the weighted average of marginal contributions and their related extensions to connect optimal credit allocation with local explanations^27^. The RF model shows good validation performance. With 20% of the data extracted for validation (Fig. 2B), it demonstrates high fidelity in estimating the observed energy consumption with R^2^ of 0.996.

Taking the advantage of RF that provides a ranking of variables based on their relative significance, the model concludes that ecological footprint, biocapacity, GDP, and population are the most important influencing factors on total energy consumption (Fig. 2A). This indicates a strong connection between energy consumption and the “planetary boundary^28^”. Although they are not variables of the planetary boundary in a traditional sense, ecological footprint and biocapacity, together, are good indications of sustainability. They are tantamount to the concepts of demand and supply in Economics. Ecological footprint is the human demand on nature, and biocapacity is the ecological supply^29^. Therefore, these two variables measure human’s interaction with the biosphere. The ratio between ecological footprint and biocapacity demonstrates an overall linear relationship with energy consumption. It grew from 0.825 in 1965 to 1.766 in 2020^30^, surpassing the double of its initial value. Besides the planetary boundary, there also exists a strong relationship between energy consumption and the economy, though the causality between them is still being debated^31^. The growth of GDP usually demands energy consumption in almost all areas, and they show approximately a linear relationship (Supplementary Fig. 1). The world’s total GDP has increased in the past few decades with an average of ~ 1.345 trillion dollars (2015 constant USD) each year from 18.05 trillion dollars in 1970 to 86.65 trillion dollars in 2021^32^. Being one of the economic indicators, population is a large contributor as well, likely due to its correlation with residential energy consumption. The global population has grown from 3.68 billion in 1970 to 7.76 billion in the past 5 decades, and it demonstrates a linear feature with an average annual increase of 81.4 million. Despite economic indicators, energy consumption shows a certain amount of connection with temperature and precipitation, as the level of which will directly lead to change in electricity consumption^33^. Results of the RF model indicate that energy consumption is closely related to human consumption of Earth’s biological resources and economic as well as climate indicators.

### Total energy consumption prediction

Global energy consumption is predicted till 2060 using the trained RF model with the projections of major contributors. Due to the lack of complete data, not all countries are considered in the prediction. A total of 40 countries that play major roles in the global economy and development, among the original 66 used for training, are included in this forecast, and the results are further processed with the contribution ratio of these 40 countries to reach the conclusion. The central concern behind this approach is that the model is trained with data from individual countries. Therefore, the prediction subject must correspond to its scale. For the forecast of temperature and precipitation, several presumed shared socioeconomic pathway scenarios (SSP) are considered in CMIP6, which describe plausible alternative trends in the evolution of society and ecosystems over a century timescale. SSP, both covering different climate futures and different possible and internally consistent socioeconomic developments, aid in our comprehension of the long-term effects of near-term decisions and allow for the investigation of many future scenarios in the context of fundamental future uncertainties^34,35^. We test our model with additional climate inputs including several greenhouse gas variables in Supplemental Information.

**Figure 3:**
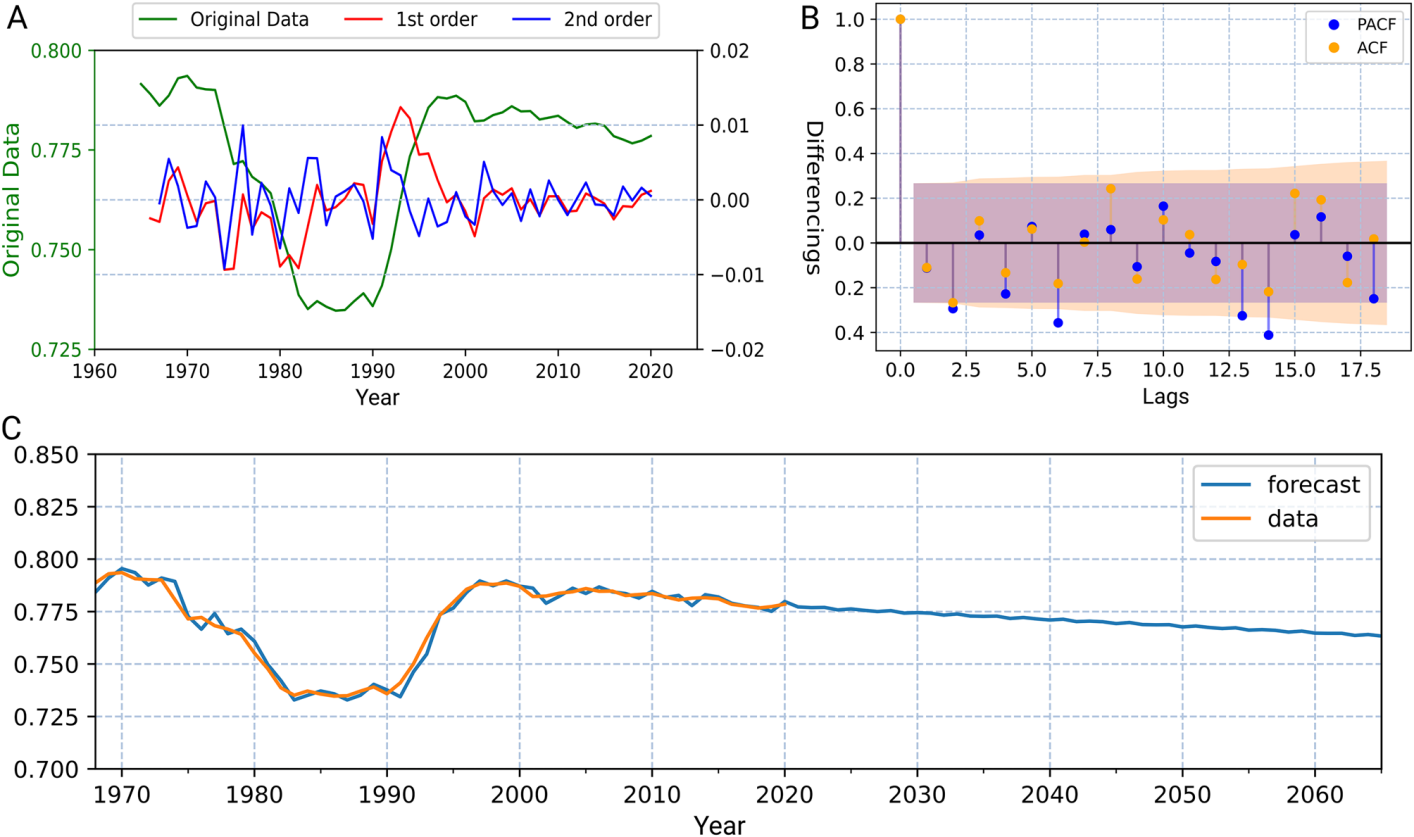
Results of the ARIMA prediction. (**A**) Three orders of differencing; the red curve displays the difference between two consecutive years of data in the original dataset, and the blue curve shows the values obtained by differencing the differenced data a second time. (**B**) Autocorrelation, which is the correlation between time series with a lagged version of itself, and partial autocorrelation, which is the additional correlation explained by each successive lagged term of the model; values beyond the shaded zone signify strong statistical significance. (**C**) The ratio of the total energy consumption (yellow curve) of the selected 42 countries to the global energy consumption together with the prediction results (blue curve).

In order to achieve the total energy consumption prediction using the results of the selected 40 countries, ARIMA model is applied to predict the proportion of the sum of these countries to the world’s total (Fig. 3). Given that the energy consumption growth of most selected countries follows their historical patterns and displays linear trends, time series modeling is an ideal tool to predict the said proportion. Results of the ARIMA show that such a ratio is projected to experience a gradual decline over the period of 2021 to 2060 from 0.777 to 0.765.

### Energy and civilization in 2060

**Figure 4:**
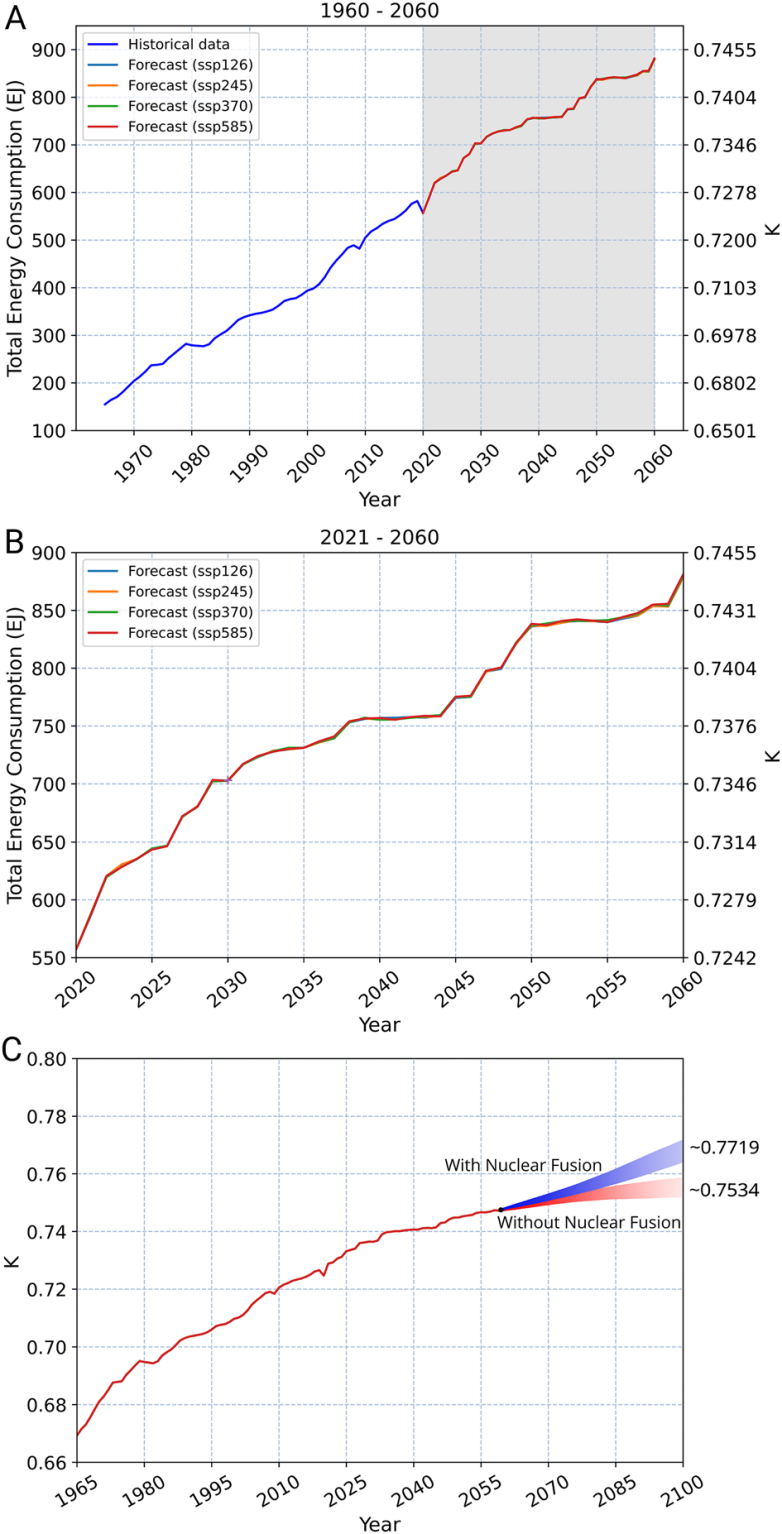
Forecast of energy consumption and index K on the Kardashev Scale. Energy consumption is predicted through four shared socioeconomic pathway scenarios (SSP): SSP126 (light blue), SSP245 (yellow), SSP370 (green), and SSP585 (red). They each represent different socioeconomic scenarios with additional radiative forcing of 2.6 W/m^2^, 4.5 W/m^2^, 7.0 W/m^2^, and 8.5 W/m^2^, respectively. (**A**) Historical data and prediction. (**B**) A zoom-in version of the shaded area of the prediction, focusing specifically on the predicted values. (**C**) The trend of the index K under two hypothetical scenarios, with (blue) and without (red) the advent of nuclear fusion in 2060, on mankind’s development.

Combining the energy consumption prediction of the selected countries and the forecasted trend of their portion, we are able to predict global energy consumption and mankind’s position on the Kardashev Scale (Fig. 4A, Table 1). The prediction shows that the world’s total energy consumption is expected to reach ~ 887 EJ (8.87 × 10^20^ J) in 2060, with a growth of over 50% within the coming 40 years. Human civilization is prospected to achieve Type 0.7449 in 2060 with a humble average annual growth rate of approximately 0.042% from 2021 to 2060, and a total growth of K = 0.0173 from 2020. Predictions with and without temperature and precipitation show negligible distinction in four prediction curves (Fig. 4B). The energy consumption displays a linear feature in the prediction, which corresponds to a decreasing slope of the index K. Since there exists a logarithmic relation between K and energy consumption, the index K will likely increase at an even slower rate in the future if there are no changes in the energy structure.

**Table 1:** Final forecasting results.

Year	Projected values		
	Energy consumption (EJ)	K	
		Value	Growth rate (past 5 years)
2025	629.07	0.72999	3.28 × 10^–3^
2030	697.28	0.73446	6.12 × 10^–3^
2035	732.89	0.73662	2.94 × 10^–3^
2040	747.90	0.73750	1.19 × 10^–3^
2045	777.81	0.73921	2.32 × 10^–3^
2050	836.92	0.74239	4.30 × 10^–3^
2055	843.36	0.74272	4.45 × 10^–4^
2060	887.14	0.74492	2.96 × 10^–3^

The predicted values for energy consumption, civilization development index K, as well as its growth rate over each 5-year period.

### Influence of the advent of nuclear fusion

To understand the potential impact of fusion energy, a scenario is examined assuming the advent of nuclear fusion around 2060, along with corresponding fast technological advancement and economic growth. Unlike other contributing factors considered in this study, in the case of nuclear fusion, no historical pattern can be accounted to foresee the future of innovations. Thus, we assume that its impact will drive the world’s energy demand with a trend similar to that of the most recent energy revolution. The impact of nuclear fusion on humanity’s development on the Kardashev Scale is assumed to be identical to that of the Second Industrial Revolution. The trend of K demonstrates a greater positive slope right after the beginning of the Second Industrial Revolution (Supplementary Fig. 2A), which indicates an exponential increase of energy consumption. Under this scenario, the total energy consumption with the advent of nuclear fusion is expected to experience similar exponential growth. With an assumption of the same increasing rate, the index K is expected to reach Type ~ 0.7719 at the end of the twenty-first century (Fig. 4C). In contrast, if the current energy structure does not change, and the human civilization follows its current trend, the index K can only be able to merely achieve Type ~ 0.7534 by the end of the twenty-first century.

## Discussion

The prediction of energy consumption is crucial in shaping the future structure of global energy consumption and potentially impacting the development of civilization^16^. The results of this study demonstrate a quantitative prediction of energy consumption. It fills the gap of mid-term projection of civilization type on the time scale of a few decades. Our results mark the first attempt to predict the future advancement of human civilization on the Kardashev Scale with multi-dimensional data taken into consideration. However, certain unquantifiable factors, such as national energy policies, may introduce additional uncertainties and challenges to energy consumption prediction^16,36^. More accurate prediction is anticipated with updated data and advanced models. Nonetheless, our application of machine learning algorithms and in-depth data mining with comprehensive datasets has enabled us to improve the precision.

Previous works also attempted to foresee humanity’s usage of energy in the future. In the 2022 bp Energy Outlook, primary energy demand is forecasted till 2050 under three different presumed scenarios including Accelerated, Net Zero, and New Momentum^37^. Using them to explore the range of possible outcomes over the next 30 years, they conclude that the energy demand might achieve ~ 660 to ~ 760 EJ in the year of 2050. Sustainability may likely be a limiting factor in the future for the increase of energy consumption. Established on the installation rate of renewable energy, another study predicted the energy demand based on the fossil fuel emissions cap, phase-out profile, and the investment return on the renewable energy^38^. Under the average scenario, the path shows that energy demand will be ~ 2.2 × 10^5^ TWh (792 EJ, Type 0.7400) in 2060.

The development of human civilization towards the Kardashev limit is not only constrained by energy transformation. Although the RF model results show environmental parameters have low impacts on energy consumption, this situation may change in the long term if the current energy structure, with a high proportion of fossil fuels up to 80%, continues. Another study argues that perceptual distortions and prevailing economic rationality accelerate the depletion of natural resources^39^. The limits of technology, resources, and exponential growth itself can also be factors that constrain the progression of mankind on the Kardashev Scale^40^. However, despite of these unignorable concerns and the unpredictability of the future, this study serves as a benchmark of energy growth under the scenario of the trends of the current economy, population, climate, and human use of natural resources.

The world’s largest fusion experiment, ITER, is set to achieve its first full-scale plasma in 2025, and the test for fusion is expected to begin a decade later^41^. After an estimated testing period of ~ 25 years, the European roadmap aims to generate net energy output and establish facilities to convert said energy to electricity^42^. Therefore, nuclear fusion, along with other promising technologies in energy, has the potential to serve as a milestone of humanity’s energy progression and transformation during the 6th decade of this century^43–45^. Although the estimated time of the advent of nuclear fusion has kept changing for decades and the development of this technology is filled with uncertainties, its influence will likely be significant once the technology becomes mature. While it is true that nuclear fission energy, currently commonly referred as nuclear energy, was regarded with similar perception and was seen as the “energy future” in the 1950s, fusion is fundamentally different. The storage of accumulated radioactive waste limits the future of nuclear fission energy^46^; fusion technology, on the other hand, has the prospective to change the world’s energy structure, and thereby start a new industrial revolution. Although there exist many inevitable challenges^47^, nuclear fusion has a promising potential to energy production by the end of the twenty-first century.

## Summary

Energy is vital to civilizations. The hidden patterns and behaviors of energy consumption and its related parameters provide clues for future energy growth. By analyzing these patterns and related parameters, the RF model can make predictions that provide valuable insights for the world energy sector to make necessary adjustments in favor of development. Through a unique approach to aiding the results with ARIMA, we conclude that in 2060, human civilization will be able to achieve Type 0.7449 on the scale with an annual energy consumption of ~ 887 EJ. Our methods significantly improve upon the previous projection of K, which oversimplifies the task. Uncertainties of the energy future and many existing concerns present a series of challenges to humanity. If the energy strategies and technologies remain in the present course, it may take human civilization millennia to become a Type 1 civilization. In order to accelerate the progression of our civilization, there are two potential pathways. The development of renewable energy prospective technologies such as carbon capture and sequestration address this issue by focusing on sustainability; nuclear fusion address the issue by targeting significant energy production advancements^48–51^. A combination of these two methods can pave the way toward sustainable and significant energy production advancements.

## Methods

### Variable selection

Developing an RF model requires reliable input parameters that are related to the targeted output. Therefore, the selection of input variables becomes a significant step. Energy consumption, which is intimately linked with human activities, closely follows economic and social factors. In order to identify the specific variables that potentially have an impact on primary energy consumption, several aspects are carefully considered. By investigating multiple international databases and reviewing related articles, we concluded four general types of variables that could potentially influence energy consumption.

The connection between energy consumption and economic growth has been confirmed by studies^52,53^. Among the economic indicators, gross domestic product (GDP) stands out as the most representative factor. Here, the constant 2015 U.S. dollars is selected as the unit of all data to eliminate potential biases caused by inflation. The World Bank provides historical GDP data by country. For forecasting input, we utilized data from Organization for Economic Co-operation and Development (OECD). Applying internationally accepted standards, both of these organizations provide reliable information as well as data sources. The causality between energy consumption and human activity is extremely strong^54^. Variables including population and urban population hold a strong correlation with energy consumption essentially because human activity directly affects the amount of energy being consumed. The training set of data is again from the World Bank. The forecast data of these variables are pulled from the United Nations (UN) data bank. UN is one of the most authoritative organizations, and its statistics division strives for the highest level of accuracy.

Energy consumption is also closely linked with various climate indicators. Studies show that GHG emission is a determinant of increasing energy consumption internationally^55,56^. However, GHG variables played no role in the economic development of human societies historically, and are, therefore, not included in the main results. Climate indicators are potentially important limiting factors in the future; presently, they show very little impact on energy consumption (Supplementary Fig. 3). Temperature and precipitation, on the other hand, have always been impact factors of energy consumption since they directly affect human demand for energy. Thus, they are selected as model inputs; additional results with carbon dioxide mole fraction, methane mole fraction, aerosol optical depth at 550 nm, and atmosphere mass content of water vapor added into consideration can be found in Supplemental Information. Both historical and forecast data of all variables have source ID(ESM) MRI-ESM2-0 and are from Coupled Model Inter-comparison Project Phase 6 (CMIP6). The most recent CMIP6 is a project of the World Climate Research Programme (WCRP)’s Working Group of Coupled Modelling (WGCM). CMIP6 model simulations have been regularly assessed as part of the IPCC Climate Assessments Reports and various national assessments.

Energy availability is not all that is required for human civilization to thrive. The term “planetary boundary” describes nine variables of high importance to the habitability of Earth, including biosphere integrity, ocean acidification, freshwater use, climate change, and so on. Yet, these variables are quite difficult to measure globally, especially when data of specific countries are needed. Inspired by using net primary production to measure planetary boundary for the biosphere^57^, we included ecological footprint and biocapacity in the model. These two factors can be quantified, and they are good indicators of human consumption of Earth’s biological resources. The data is sourced from York University, Ecological Footprint Initiative.

Identification factor ISO 3166 country code is also incorporated as a model input. Although it does not show a direct correlation with energy consumption, it distinguishes the countries, which might actively play a role in the prediction process.

### Random forest

RF is a decision tree-based machine learning approach. It bootstraps each tree and splits each point in the tree into subsets based on the best of a subset of randomly picked predictors at each point. RF has some advantages over typical parametric modeling techniques. It is capable of handling non-linear relationships with a low risk of overfitting, and it is also able to handle highly correlated variables. In comparison to parametric approaches, it also has a higher prediction accuracy and provides information about the underlying mechanism in the form of variable relevance of the predictor variables^58^.

To further stabilize the algorithm and improve performance in general, the model is trained with the gradient boosted decision trees^59^ (GBDT). The general idea of “boosting” targets to covert weak learning algorithms to ones that reach arbitrarily high accuracy. It functions by sequentially applying weak learners to consistently re-weighted versions of the training data. Through the boosting iterations, classifiers focus on values that have been hard to classify in past steps by increasing the weight of misclassified values and decreasing the weight of correctly classified values. Subsequently, the predictions of the series of weak classifiers are merged by a weighted majority vote into a final projection.

Python package LightGBM^60^ is used to perform the calculation.

### Autoregressive integrated moving average

Projected values of the ARIMA models are presumed as linear functions of past data and random errors. The time series’ standard formulation of the underlying process can be represented by equation^61^

$${\upgamma }_{t}={\theta }_{0}+{\theta }_{1}{\gamma }_{t-1}+{\theta }_{2}{\gamma }_{t-2}+\dots +{\theta }_{p}{\gamma }_{t-p}+ {\beta }_{1}{\varepsilon }_{t-1}+{\beta }_{2}{\varepsilon }_{t-2}+\dots +{\beta }_{p}{\varepsilon }_{t-p}$$

where ${\upgamma }_{t}$ represents the future value, and indexes p and q, respectively, are commonly referred as the order for AR and MA part of the model. ${\theta }_{i}{\gamma }_{t-i}$, ${\beta }_{i}{\varepsilon }_{t-i}$ are the model parameters where past values ${\upgamma }_{t-i}\left(i=1, 2, \dots , p\right)$ and random errors ${\upvarepsilon }_{t-i}\left(i=1, 2, \dots , q\right)$ are multiplied by distinct coefficients ${\theta }_{i}$ and ${\beta }_{i}$, respectively. Further, ${\varepsilon }_{t}$ are assumed to be independently and identically distributed with a mean of zero and a constant variance of ${\sigma }^{2}$.

### Impact of nuclear fusion

Presuming that the advent of nuclear fusion will have similar impact on global energy consumption, we calculated the potential pathway humanity might undertake with the influence of fusion energy. By extending the linear trend presented in the energy consumption prediction from 2051 to 2060, the civilization Type of humanity at the end of the century is approximated. Linear regression is applied to fit the trends of K before and after the beginning of the Second Industrial Revolution, and the slopes of the fitting lines appear to be 4.94 × 10^–4^ and 9.46 × 10^–4^, respectively. Using the prediction results, we further approximated the growth of K with the impact of nuclear fusion by adding the hypothetical growth, which can be calculated by multiplying the length of the period and the difference of the computed slopes, onto the approximated value of K in 2100.

## Supplementary Information

Supplementary Information.

## Data Availability

Training data for economic indicators and demographic parameters are provided by World Bank, GDP, population, urban population, and urbanization; data for climate variables are from Coupled Model Inter-comparison Project Phase 6 (CMIP6) (https://esgf-node.llnl.gov/projects/cmip6/), including temperature and precipitation; data for ecological footprint and biocapacity is from York University (https://footprint.info.yorku.ca/data/). The final datasets used for training and forecasting are uploaded (https://github.com/AntongZ1/Data). Predictions of GDP, population variables, and climate variables are provided by the OECD (https://data.oecd.org/gdp/gdp-long-term-forecast.htm), UN (https://population.un.org/wpp/), and CMIP6, respectively.
