# Supplementary material for: Forecasting the progression of human civilization on the Kardashev Scale through 2060 with a machine learning approach
Source: Sci Rep. 2023 Jul 12;13:11305. doi: 10.1038/s41598-023-38351-y (PMC10338519; doi:10.1038/s41598-023-38351-y)
Supplement: Supplementary file 1 — Supplementary Information. [file 41598_2023_38351_MOESM1_ESM.pdf]

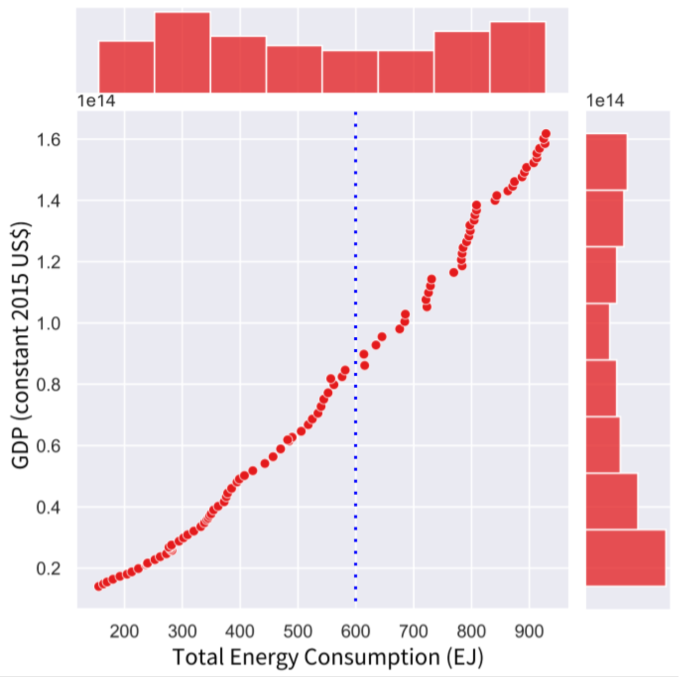

Supplementary Figure 1. Direct correlation of world's GDP and energy consumption. The joint plot that describes the correlation of GDP and energy consumption is graphed, where the left of the blue dotted line is historical data, and the right is projected values.

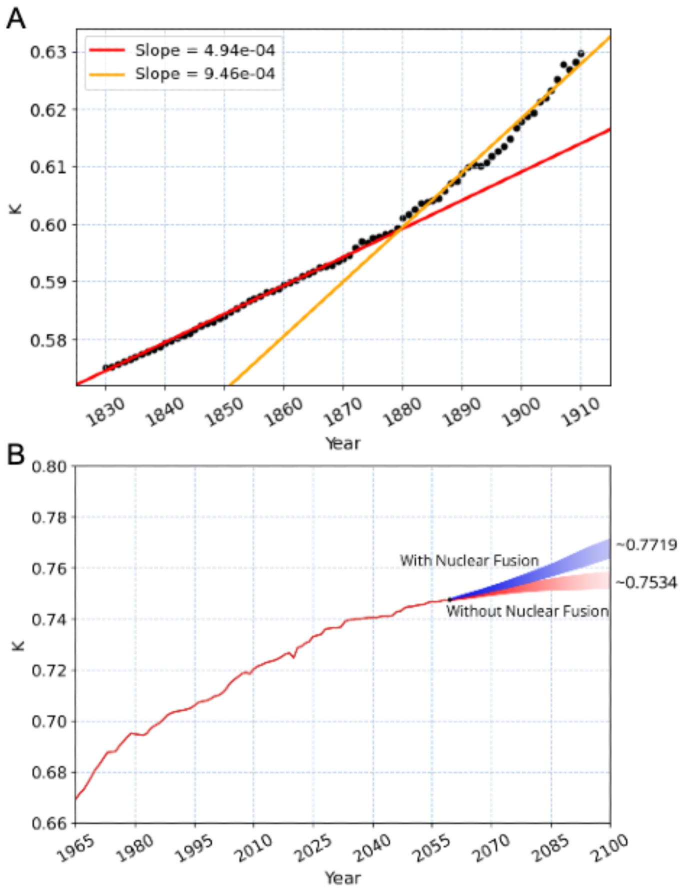

Supplementary Figure 2. Potential impact of fusion energy. (A) The trend of K before and after the Second Industrial Revolution. The red and orange lines present the linear regression trending line of K before and after the Second Industrial Revolution, respectively. (B) The trend of the index K under two hypothetical scenarios, with (blue) and without (red) the advent of nuclear fusion in 2060, on mankind's development.

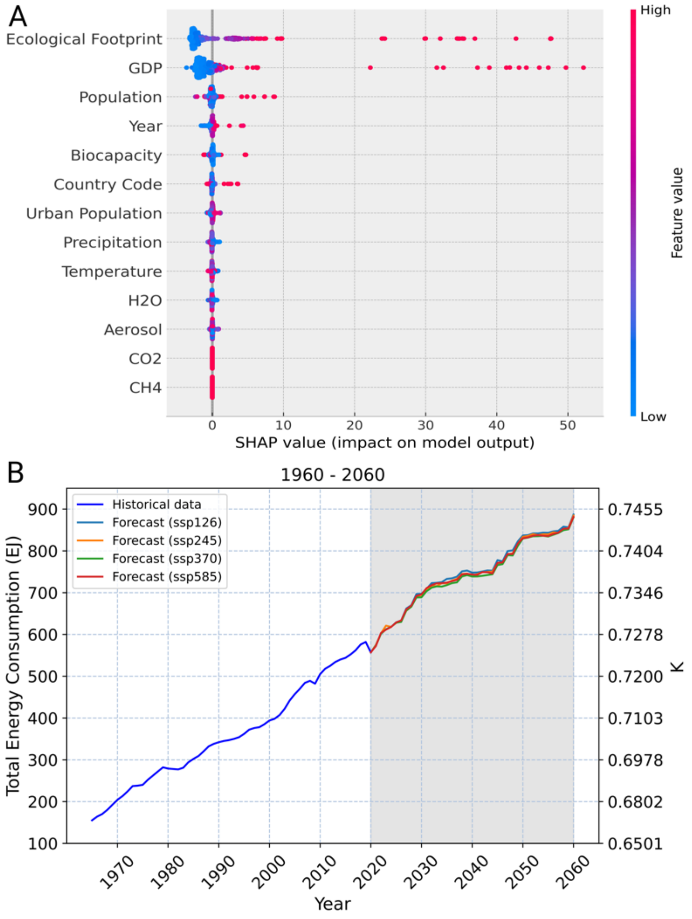

Supplementary Figure 3. Forecast of energy consumption and index K on the Kardashev Scale with additional impact factors (atmosphere mass content of water vapor, aerosol optical depth at 550nm, carbon dioxide mole fraction, and methane mole fraction) in the model. (A) The selected factors' impacts on the total energy consumption are measured by their SHAP values. The color of each individual dot represents the value of that feature. (B) Historical data and prediction.

Year	Projected Values					
	Energy Consumption (EJ)				K	
	SSP126	SSP245	SSP370	SSP585	Value	Annual Growth Rate
**2025**	643.96	643.61	644.39	643.44	0.73101	4.69×10^{-3}
**2030**	702.96	702.98	702.52	702.58	0.73481	5.20×10^{-3}
**2035**	731.15	731.14	731.19	731.07	0.73652	2.33×10^{-3}
**2040**	756.97	755.43	755.43	756.91	0.73803	2.05×10^{-3}
**2045**	774.31	775.25	775.46	775.24	0.73901	1.33×10^{-3}
**2050**	836.22	836.53	836.74	838.37	0.74235	4.52×10^{-3}
**2055**	839.86	840.74	841.56	839.84	0.74254	2.56×10^{-4}
**2060**	879.43	878.70	879.82	881.20	0.74454	2.69×10^{-3}

Supplementary Table 1. **Forecasting results with additional impact factors (atmosphere mass content of water vapor, aerosol optical depth at 550nm, carbon dioxide mole fraction, and methane mole fraction) in the model.** The predicted value for energy consumption under four different presumed shared socioeconomic pathway scenarios, civilization development index K, as well as its growth rate from 2021 to 2060.

Parameter	Source	
	**Training**	**Forecasting**
Energy Consumption	bp Global	/
GDP	World Bank	Organization for Economic Co-operation and Development (OECD)
Population	World Bank	United Nation (UN)
Urban Population	World Bank	United Nation (UN)
Ecological Footprint	York University	ARIMA Forecasting
Biocapacity	York University	ARIMA Forecasting
Temperature	Coupled Model Inter-comparison Project Phase 6 (CMIP 6)	
Precipitation	Coupled Model Inter-comparison Project Phase 6 (CMIP 6)	
Atmosphere Mass Content of Water Vapor	Coupled Model Inter-comparison Project Phase 6 (CMIP 6)	
Aerosol Optical Depth (500nm)	Coupled Model Inter-comparison Project Phase 6 (CMIP 6)	
Carbon Dioxide Mole Fraction	Coupled Model Inter-comparison Project Phase 6 (CMIP 6)	
Methane Mole Fraction	Coupled Model Inter-comparison Project Phase 6 (CMIP 6)	

Supplementary Table 2. **Data sources.**
